# Urinary concentrations of phthalate biomarkers and weight change among postmenopausal women: a prospective cohort study

**DOI:** 10.1186/s12940-019-0458-6

**Published:** 2019-03-12

**Authors:** Mary V. Díaz Santana, Susan E. Hankinson, Carol Bigelow, Susan R. Sturgeon, R. Thomas Zoeller, Lesley Tinker, Jo Ann E. Manson, Antonia M. Calafat, Jaymie R. Meliker, Katherine W. Reeves

**Affiliations:** 1 Department of Biostatistics and Epidemiology, University of Massachusetts Amherst, 411 Arnold House, 715 North Pleasant Street, Amherst, MA 01003 USA; 2 Department of Biology, University of Massachusetts Amherst, Amherst, MA USA; 3 Cancer Prevention Program, Fred Hutchinson Cancer Research Center, Seattle, WA USA; 4 Department of Medicine, Brigham and Women’s Hospital, Harvard Medical School, and the Harvard T.H. Chan School of Public Health, Boston, MA USA; 5 Division of Laboratory Sciences, National Center for Environmental Health, Centers for Disease Control and Prevention, Atlanta, GA USA; 6 Program in Public Health, Department of Family Population and Preventive Medicine, Stony Brook University, Stony Brook, USA

**Keywords:** Phthalates, Endocrine disruption, Obesity, Weight change, Postmenopause, Women

## Abstract

**Background:**

Some phthalates are endocrine disrupting chemicals used as plasticizers in consumer products, and have been associated with obesity in cross-sectional studies, yet prospective evaluations of weight change are lacking. Our objective was to evaluate associations between phthalate biomarker concentrations and weight and weight change among postmenopausal women.

**Methods:**

We performed cross-sectional (*N* = 997) and longitudinal analyses (*N* = 660) among postmenopausal Women’s Health Initiative participants. We measured 13 phthalate metabolites and creatinine in spot urine samples provided at baseline. Participants’ weight and height measured at in-person clinic visits at baseline, year 3, and year 6 were used to calculate body mass index (BMI). We fit multivariable multinomial logistic regression models to explore cross-sectional associations between each phthalate biomarker and baseline BMI category. We evaluated longitudinal associations between each biomarker and weight change using mixed effects linear regression models.

**Results:**

In cross-sectional analyses, urinary concentrations of some biomarkers were positively associated with obesity prevalence (e.g. sum of di (2-ethylhexyl) phthalate metabolites [ΣDEHP] 4th vs 1st quartile OR = 3.29, 95% CI 1.80–6.03 [p trend< 0.001] vs normal). In longitudinal analyses, positive trends with weight gain between baseline and year 3 were observed for mono-(2-ethyl-5-oxohexyl) phthalate, monoethyl phthalate (MEP), mono-hydroxybutyl phthalate, and mono-hydroxyisobutyl phthalate (e.g. + 2.32 kg [95% CI 0.93–3.72] for 4th vs 1st quartile of MEP; p trend < 0.001). No statistically significant associations were observed between biomarkers and weight gain over 6 years.

**Conclusions:**

Certain phthalates may contribute to short-term weight gain among postmenopausal women.

**Electronic supplementary material:**

The online version of this article (10.1186/s12940-019-0458-6) contains supplementary material, which is available to authorized users.

## Introduction

The potential contribution of environmental factors to obesity is of increasing interest. “Obesogens” describes environmental chemicals hypothesized to promote obesity due to altered regulation of adipogenesis and lipid metabolism. Phthalates are endocrine disrupting chemicals present in many consumer products (e.g. cosmetics, food packaging, medications) and are ubiquitous in the environment. Nearly all U.S. residents have detectable concentrations of phthalate metabolites in their urine, though concentrations vary widely [1]. Limited in vitro data suggest that certain phthalates may alter pathways that promote adipogenesis [2, 3], and thus could impact development of obesity.

Scant research evaluating associations between phthalate exposure and body weight report inconsistent findings. Some cross-sectional studies report positive associations between certain phthalate metabolite concentrations and body mass index (BMI) and obesity among adult women. Specifically, one cross-sectional study using data from the 2007–2010 National Health and Nutrition Examination Survey (NHANES) reported increased prevalence of obesity associated with higher concentrations of mono-carboxyoctyl phthalate (MCOP), mono (2-ethyl-5-carboxypentyl) phthalate (MECPP), mono (2-ethyl-5-hydroxyhexyl) phthalate (MEHHP), and the sum of di-ethylhexyl phthalate metabolites (ΣDEHP) [4]. A separate cross-sectional study using 1999–2004 NHANES data observed increased obesity prevalence associated with mono (2-ethylhexyl) phthalate (MEHP) and mono-butyl phthalate (MBP), yet found borderline significant inverse associations between MECPP, MEHHP, mono (2-ethyl-5-oxohexyl) phthalate (MEOHP), ΣDEHP and BMI category [5]. Also, other studies, including one using 1999–2002 NHANES data [6] and another within the Nurses’ Health Study (NHS) and NHS2 cohorts [7], reported inverse cross-sectional associations with MEHP [6], MBP [6, 7], mono-benzyl phthalate (MBzP) [7], and mono-isobutyl phthalate (MiBP) [7].

Phthalates are rapidly metabolized in the body and excreted in urine, and urinary phthalate metabolite concentrations reflect recent exposures [8]. Therefore, the observed cross-sectional associations may reflect confounding via exposure from sources that are themselves associated with obesity, as opposed to causal associations.

One prior prospective analysis, among 977 women aged 32–79 from the Nurses’ Health Study (NHS) and NHS2 [7], reported positive associations with weight gain for MBzP (+ 0.42 kg/year for 4th vs 1st quartiles) and the sum of butyl phthalate metabolites (MBP and MiBP; + 0.34 kg/year for 4th vs 1st quartiles) over a 10 year follow-up period. Weight change was not associated with concentrations of ΣDEHP or mono-ethyl phthalate (MEP) [7].

Whether phthalates affect weight gain remains an unanswered, yet critically important, question. We prospectively evaluated associations between 13 phthalate metabolites (or their sums) and weight change among 997 postmenopausal women enrolled in the Women’s Health Initiative (WHI).

### Subjects and methods

#### Study population

**Fig. 1:**
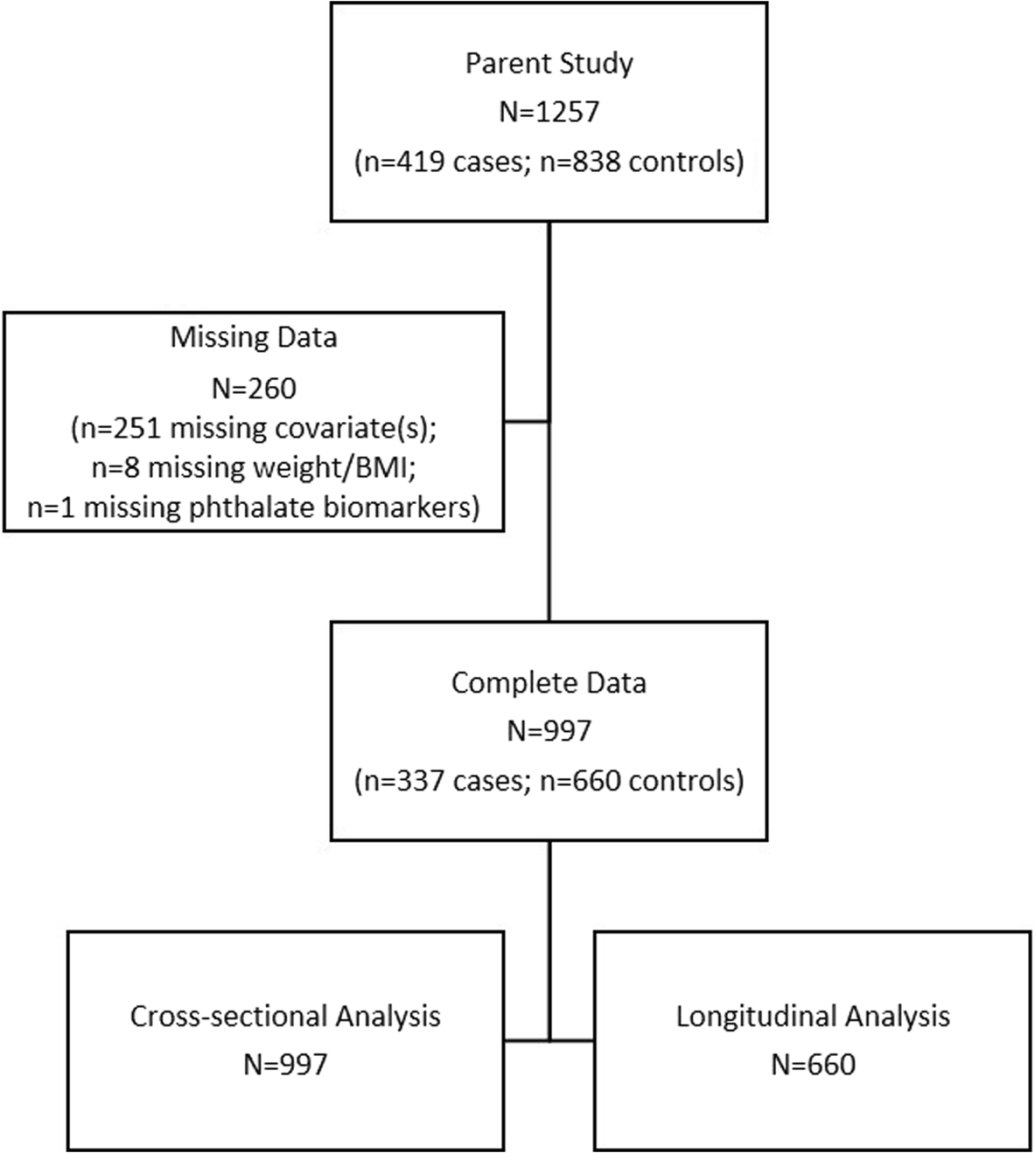
Selection of study population for the analysis of phthalate biomarkers and weight change in postmenopausal women

We included 1257 postmenopausal women selected for a nested case-control study of phthalates and breast cancer risk within the WHI. The design of the WHI has been reported previously [9]. Briefly, from October 1, 1993 to December 21, 1998 a total of 161,808 women aged 50–79 years were enrolled in the WHI. WHI participants who were enrolled at three bone density substudy sites (Birmingham, AL; Pittsburgh, PA; Tucson/Phoenix, AZ) provided first morning void urine samples at baseline. A nested case-control study of breast cancer within the WHI quantified urinary concentrations of phthalate metabolite on 419 incident breast cancer cases and 838 matched controls selected from among these bone density substudy participants. Breast cancer cases were selected as all cases of invasive breast carcinoma that occurred among these participants after the year 3 follow-up clinic visit through 2013; controls were matched on enrollment date, length of follow-up, age at enrollment, and WHI study arm with a 1:2 ratio. This analysis includes 997 participants (337 cases, 660 controls) with complete data available (Fig. 1). The longitudinal analysis included only participants selected as controls (*N* = 660) in the parent study, given that weight gain is common following breast cancer treatment [10].

All participants provided written informed consent upon enrollment into the WHI. The WHI was approved by institutional review boards (IRB) at each clinical center. Additionally IRB approval for the present study was obtained from the University of Massachusetts Amherst. The involvement of the Centers for Disease Control and Prevention (CDC) laboratory in the analysis of samples did not constitute engagement in human subjects research.

#### Quantification of urinary phthalate metabolites

WHI followed a standard collection, processing, and storage protocol at the three clinical centers that collected urine samples. First morning void urine samples were collected at home and processed within 30 min after participants arrived at the clinic. WHI recommended, but did not require, the use of phthalate-free polypropylene urine collection containers; one site used the recommended containers while the composition of the containers used at the other two clinical centers is unknown. However, all sites used polypropylene centrifuge tubes and cryovials for long-term storage. Additionally, we measured concentrations of metabolites as opposed to the parent phthalate, which should reflect endogenous exposure as opposed to contamination. Urine samples were centrifuged for 5 min at 1330×g and 1.8 mL aliquots were frozen and shipped, packed in dry ice, via overnight FedEx to McKesson Bioservices where they were stored at − 70 °C.

Thirteen phthalate metabolites were measured in baseline urine samples at the CDC: MEP, MBP, mono-hydroxybutyl phthalate (MHBP), MiBP, mono-hydroxyisobutyl phthalate (MHiBP), MBzP, mono (3-carboxypropyl) phthalate (MCPP), MEHP, MEHHP, MEOHP, MECPP, MCOP, and mono-carboxynonyl phthalate (MCNP). Concentrations of phthalate metabolites were quantified after enzymatic hydrolysis of the conjugated metabolites followed by on-line solid phase extraction coupled to high performance liquid chromatography-electrospray ionization-isotope dilution tandem mass spectrometry. Complete details of the analytical method are published online at https://wwwn.cdc.gov/nchs/data/nhanes/2013-2014/labmethods/PHTHTE_H_MET_Phthalates.pdf. The limits of detection (LODs) were in the low ng/mL range. Study samples were randomly distributed through the analytical batches, with cases and matched controls analyzed together. A blinded 10% quality control sample was included, and estimated CVs were as follows: MBP 5.4%, MBzP 6.1%, MCNP 4.7%, MCOP 6.3%, MCPP 5.8%, MECPP 4.3%, MEHHP 5.4%, MEHP 19.5%, MEOHP 6.0%, MEP 3.1%, MHBP 9.0%, MHiBP 21.9%, MiBP 10.3%; the higher average CVs for MEHP and MHiBP reflect small differences in absolute levels of replicates having very low concentrations. All laboratory staff were masked to the identity, disease status, and demographic and risk factor characteristics of the samples. Creatinine was also measured by using an enzymatic assay at CDC on a Roche Modular P Chemistry Analyzer (Indianapolis, IN). The LOD for creatinine was 10 mg/L and the CV of the blinded quality control sample was 2.5%.

We analyzed concentrations of each phthalate metabolite individually. For phthalates with multiple measured metabolites, we also grouped the data by parent phthalate by dividing each metabolite of a single parent by its molecular weight and then summing across metabolites [11, 12]. For example, we calculated the molar sum of DEHP metabolites (ΣDEHP) by dividing each metabolite concentration by its molar mass and then summing the individual concentrations (μmol/L): [MEHHP × (1/294.35)] + MEHP × (1/278.34)] + [MECPP × (1/308.33)] + [MEOHP × (1/292.33)]. The sum of dibutyl phthalate metabolites (ΣDBP) was calculated as the molar sum of MBP and MHBP, and the sum of di-isobutyl phthalate metabolites (ΣDiBP) was calculated as the molar sum of MiBP and MHiBP.

#### Measurement of weight and BMI calculation

Height and weight were measured at the baseline, year 3, and year 6 clinic visits and used to calculate BMI as weight (kg)/height^2^ (m^2^) grouped as: underweight/normal weight (< 25.0 kg/m^2^), overweight (25.0– < 30.0 kg/m^2^), and obese (≥30.0 kg/m^2^).

#### Assessment of covariates

Extensive data on demographic, reproductive, medical history, and behavioral characteristics were collected in the WHI using self-administrated questionnaires at baseline. We considered the following variables as covariates: age (continuous), race/ethnicity (Caucasian, African American, Hispanic/Latino, other), education level (<high school, high school/some college, college degree and higher), income (<$10,000, $10,000-$19,999, $20,000-$34,999, $35,000-$49,999, ≥ $50000), health insurance (no insurance, military insurance, Medicare, Medicaid, private insurance), smoking status (never smoker, past smoker, current smoker), alcohol use (non-drinker, past drinkers, current drinkers), Healthy Eating Index-2005 (HEI-2005, [13]) score (continuous), dietary energy intake (kcal per day; continuous), total recreational physical activity (categorized in quartiles of Metabolic Equivalent values per week (METs/wk.; < 1.25 METs/wk., [1.25- < 6.38 METs/wk., 6.38- < 16.5 METs/wk., ≥16.5 METs/wk), oral contraceptive use (ever, never), any hormone therapy use (never, past, current), ever had diabetes (no, yes), ever had cardiovascular disease (no, yes), hypertension (never hypertensive, untreated hypertensive, treated hypertensive), and dyslipidemia (no, yes).

### Statistical analyses

Phthalate biomarker concentrations were natural log transformed to improve normality. Baseline characteristics were summarized according to the BMI categories and differences assessed using analysis of variance (ANOVA) or chi square tests, as appropriate. Geometric means were calculated for each creatinine-standardized phthalate biomarker (i.e. individual metabolite or sum of metabolites of a common parent phthalate) with stratification on baseline BMI group, and differences across groups were assessed with ANOVA.

In cross-sectional analyses we included both cases and controls, given that cases were all diagnosed following the year 3 clinic visit and thus were considered “healthy” at baseline. We categorized phthalate metabolite concentrations into quartiles using the distribution among the controls. Linear regression and multinomial logistic regression analyses were used to model the relationship of each individual phthalate biomarker and baseline weight and BMI category, respectively. All models were adjusted for age and urinary creatinine concentration. We built the regression models by 1) fitting univariable linear and multinominal logistic regression models for each variable with weight and BMI, respectively, 2) including all variables with *p* < 0.25 in the univariable model in a preliminary multivariable model along with the phthalate biomarker, and 3) evaluating the significance of each covariate using backward selection and retaining all covariates with a *p* value < 0.10 or of known biological importance. A common set of covariates was included in the multivariable models to facilitate comparisons across phthalate biomarkers. Trends in the weight β coefficient and the odds ratio (OR) of overweight and obesity with increasing categories of phthalate biomarker were evaluated by testing the significance of a continuous variable including the median concentration of each biomarker quartile in the regression model. We included 997 participants with complete data on covariates, exposure, and outcomes in our analysis.

We modeled the prospective weight change rate over 3 and 6 years by the quartiles of urinary phthalate biomarker concentrations using mixed-effect models with: a random coefficient, a fixed effect for weight and year of follow up, and including product terms between phthalate biomarkers and year of follow up (i.e. year 3 and year 6). A parsimonious multivariable model was built using the process described above. Analyses were repeated with stratification on baseline BMI to evaluate possible effect modification, and we plotted predicted weight change over time by BMI category for models including an interaction with BMI and a model without this term. We obtained *p*-values for linear trends by including an interaction term between each year of follow up and the median concentration of each biomarker quartile in the mixed-models as a continuous variable. We considered a *P*-value < 0.05 as statistically significant. All analyses were conducted using SAS version 9.4 (SAS Institute, Cary, North Carolina) and Stata version 15.0 (Stata Corp, College Station, TX).

## Results

Table 1 shows baseline characteristics of the study population by BMI category. Compared to underweight/normal weight women, obese women were older, and more likely to be Black/African American, earn <$20,000/year, and have lower educational attainment. Obese women also were less likely to drink alcohol, had lower diet quality and higher dietary energy intake, had lower physical activity, and had more hypertension and diabetes than underweight/normal weight women.

**Table 1:** Baseline characteristics of study population by body mass index category, *N*=997

Characteristics	Underweight/Normal *N*=329	Overweight *N*=357	Obese *N*=311
Age, years; Mean (SD)	63.0 (7.2)	62.9 (6.8)	61.7 (6.5)
Height (cm); Mean (SD)	162.4 (6.1)	162.0 (6.1)	161.1 (6.0)
Weight (kg); Mean (SD)	59.9 (6.2)	71.7 (6.2)	91.0 (13.3)
Healthy eating index score; Mean (SD)	69.1 (10.2)	67.7 (10.8)	64.2 (11.6)
Dietary energy intake, kcal; Mean (SD)	1,509 (622.4)	1,657 (627.0)	1,824 (816.6)
Race/Ethnicity; N (%)			
White	293 (89.1)	301 (84.3)	235 (75.6)
Black or African American	19 (5.6)	34 (9.5)	57 (18.3)
Hispanic/Latino	8 (2.4)	18 (5.0)	14 (4.5)
Other	9 (2.7)	4 (1.1)	5 (1.6)
Income; N (%)			
< $10 000	8 (2.4)	14 (3.9))	26 (8.4)
$10 000-$19 999	39 (11.9)	38 (10.6)	59 (19.0)
$20 000-$34 999	79 (24.0)	119 (33.3)	90 (28.9)
$35 000-$49 999	66 (20.1)	75 (21.0)	54 (17.4)
≥ $50 000	127 (38.6)	102 (28.6)	68 (21.9)
Don't know	10 (3.0)	9 (2.5)	14 (4.5)
Educational level; N (%)			
High School diploma or less	67 (20.4)	95 (26.6)	106 (34.1)
Post-high school diploma/some college	104 (31.6)	129 (36.1)	121 (38.9)
College degree or more	158 (48.0)	133 (37.3)	84 (27.0)
Smoking status; N (%)			
Never smoked	179 (54.4)	206 (57.7)	177 (56.9)
Past smokers	127 (38.6)	126 (35.3)	124 (39.9)
Current smokers	23 (7.0)	25 (7.0)	10 (3.2)
Alcohol use; N (%)			
Non-drinkers	35 (10.7)	46 (12.9)	57 (18.4)
Past drinkers	46 (14.0)	70 (19.6)	75 (24.2)
Drinkers	247 (75.3)	241 (67.5)	178 (57.4)
Recreational physical activity, METs/wk; N (%)			
< 1.25	52 (15.8)	99 (27.7)	110 (35.4)
1.25-6.38	85 (25.8)	76 (21.3)	93 (29.9)
6.38-16.5	92 (28.0)	88 (24.7)	57 (18.3)
≥ 16.5	100 (30.4)	94 (26.3)	51 (16.4)
Hormone therapy use; N (%)			
Never	128 (38.9)	160 (44.8)	172 (55.3)
Past user	41 (12.5)	51 (14.3)	40 (12.9)
Current user	160 (48.6)	146 (40.9)	99 (31.8)
Hypertension; N (%)			
Never hypertensive	257 (78.1)	248 (69.5)	169 (54.3)
Untreated hypertensive	19 (5.8)	25 (7.0)	28 (9.0)
Treated hypertensive	53 (16.1)	84 (23.5)	114 (36.7)
Dyslipidemia; N (%)	34 (10.3)	49 (13.7)	36 (11.6)
Cardiovascular disease; N (%)	47 (14.3)	47 (13.2)	60 (19.3)
Diabetes; N (%)	6 (1.8)	13 (3.6)	32 (10.3)

Table 2 displays the creatinine-corrected geometric mean concentrations and 95% confidence intervals for each phthalate metabolite by BMI category. MECPP and MEHHP concentrations were significantly higher among obese and overweight women compared to underweight/normal weight women, although the difference in means was small. MiBP concentrations were observed to be slightly higher among obese and overweight women compared to underweight/normal weight women. The geometric means of the other measured metabolites did not significantly differ by BMI category.

**Table 2:** Geometric mean of urinary concentrations of phthalate metabolites (ng/mg creatinine) by categories of body mass index at baseline, *N*=997

Phthalate Metabolites (ng/mg creatinine)	Underweight/Normal *N*=329	Overweight *N*=357	Obese *N*=311	*P* Value
	Geometric Mean (95% CI)			
MEP	147.4 (131.6-165.0)	132.8 (119.0-148.2)	132.2 (117.5-148.7)	0.96
MBP	37.9 (35.1-40.9)	39.4 (36.4-42.7)	36.2 (33.4-39.2)	0.19
MHBP	3.46 (3.18-3.76)	3.20 (2.96-3.46)	2.69 (2.46-2.93)	0.99
MiBP	2.92 (2.69-3.17)	3.56 (3.25-3.89)	3.27 (2.99-3.57)	0.03
MHiBP	1.44 (1.35-1.53)	1.49 (1.38-1.61)	1.23 (1.14-1.32)	0.04
MBzP	16.5 (15.3-17.8)	18.5 (17.3-19.9)	18.7 (17.1-20.2)	0.29
MCPP	4.68 (4.37-5.01)	4.89 (4.62-5.16)	5.11 (4.74-5.50)	0.20
MEHP	3.1 (2.87-3.43)	3.6 (3.28-3.97)	3.20 (2.92-3.52)	0.12
MEHHP	25.2 (23.3-27.2)	31.7 (29.3-34.3)	31.6 (29.4-33.9)	0.05
MEOHP	15.9 (14.7-17.1)	19.5 (18.0-21.1)	19.1 (17.8-20.6)	0.08
MECPP	32.7 (30.6-35.0)	41.1 (38.3-44.2)	41.9 (39.2-44.8)	0.02
MCOP	5.55 (5.15-5.98)	6.71 (6.21-7.24)	6.77 (6.29-7.28)	0.17
MCNP	4.40 (4.07-4.77)	4.84 (4.51-5.19)	5.06 (4.66-5.49)	0.24

Abbreviations used: *CI* confidence interval, *MEP* monoethyl phthalate, *MBP* monobutyl phthalate, *MHBP* mono-hydroxybutyl phthalate, *MiBP* mono-isobutyl phthalate, *phthalate MHiBP* mono-hydroxyisobutyl phthalate, *MBzP* monobenzyl phthalate, *MCPP* mono(3-carboxypropyl) phthalate, *MEHP* mono(2-ethylhexyl) phthalate, *MEHHP* mono(2-ethyl-5-hydroxyhexyl) phthalate, *MEOHP* mono(2-ethyl-5-oxohexyl), *MECPP* mono(2-ethyl-5-carboxypentyl) phthalate, *MCOP* mono-carboxyoctyl phthalate, *MCNP* mono-carboxynonyl phthalate

Table 3 displays cross-sectional associations between urinary phthalate biomarker concentrations and BMI category. In multivariable adjusted multinomial logistic regression models, we observed statistically significant positive trends in association between quartiles of MiBP, MCNP, MCOP, MCPP, ΣDEHP, and the DEHP metabolites MECPP, MEHHP, and MEOHP. The association between ΣDEHP and BMI category was particularly strong, with those in the 4th quartile of ΣDEHP having two-fold increased odds of overweight (OR 2.72, 95% CI 1.57–4.72) and three-fold increased odds of obesity (OR 3.29, 95% CI 1.80–6.03) compared to those in the 1st quartile. Obesity was positively associated with MBzP (OR 2.73, 95% CI 1.48–5.04 for 4th vs 1st quartile; p trend = 0.01), though the association of overweight was attenuated (OR 1.58, 95% CI 0.94–2.66; p trend = 0.18). MEP and MHBP concentrations were inversely associated with obesity, although the association with MHBP was not statistically significant (p trend = 0.04 and p trend = 0.10, respectively). Analyses with weight as the outcome showed similar trends, as expected (Additional file 1: Table S1). We observed similar associations when restricting to participants selected as controls (Additional file 1: Tables S2 and Table S3). We reran analyses excluding the four variables with the highest amounts of missing data as covariates in the model (physical activity, hypertension, high cholesterol, and cardiovascular disease history); results were generally similar for all phthalate biomarkers in this larger sample population (*N* = 1187) (Additional file 1: Table S4).

**Table 3:** Cross-sectional associations between phthalate biomarker concentrations and overweight and obesity compared to underweight/normal within the Women’s Health Initiative (*N*=997)

Phthalate biomarker, ng/mL	Overweight OR (95% CI)		Obese OR (95% CI)	
	Model 1^a^	Model 2^b^	Model 1^a^	Model 2^b^
MEP				
2.80 - 33.10	ref	ref	ref	ref
33.20 - 67.90	0.98 (0.64-1.51)	1.01 (0.64-1.58)	0.79 (0.50-1.24)	0.71 (0.42-1.19)
68.10 - 159.00	0.91 (0.58-1.43)	0.85 (0.53-1.37)	0.81 (0.51-1.28)	0.64 (0.38-1.08)
161.00 - 26000.00	0.86 (0.54-1.35)	0.80 (0.50-1.28)	0.71 (0.45-1.14)	0.56 (0.33-0.96)
P trend	0.48	0.29	0.20	0.04
MBP				
0.28 - 12.00	ref	ref	ref	ref
12.10 - 23.60	1.06 (0.69-1.64)	1.12 (0.72-1.77)	1.12 (0.70-1.80)	1.28 (0.75-2.17)
23.70 - 46.70	1.05 (0.67-1.65)	1.12 (0.70-1.79)	1.37 (0.86-2.21)	1.55 (0.90-2.64)
46.80 - 3600.00	0.82 (0.51-1.33)	0.94 (0.57-1.56)	0.89 (0.54-1.48)	1.11 (0.63-1.97)
P trend	0.45	0.81	0.78	0.64
MHBP				
0.28 - 0.90	ref	ref	ref	ref
1.00 - 1.90	1.02 (0.66-1.57)	1.04 (0.66-1.64)	0.91 (0.57-1.44)	1.01 (0.60-1.69)
2.00 - 3.90	0.86 (0.55-1.36)	0.94 (0.59-1.51)	0.94 (0.59-1.50)	1.11 (0.65-1.90)
4.00 - 490.00	0.57 (0.36-0.91)	0.63 (0.39-1.03)	0.46 (0.28-0.75)	0.60 (0.34-1.05)
P trend	0.02	0.07	0.004	0.10
ΣDBP, μmol/L				
0.002 - 0.065	ref	ref	ref	ref
0.0652 - 0.132	1.01 (0.67-1.53)	1.06 (0.69-1.63)	1.19 (0.76-1.87)	1.25 (0.76-2.07)
0.133 - 0.264	0.89 (0.57-1.39)	0.97 (0.61-1.53)	1.22 (0.77-1.94)	1.40 (0.84-2.36)
0.265 - 18.255	0.84 (0.52-1.37)	0.93 (0.56-1.56)	0.92 (0.55-1.54)	1.13 (0.63-2.01)
P trend	0.45	0.76	0.86	0.56
MiBP				
0.14 - 1.00	ref	ref	ref	ref
1.10 - 2.10	1.13 (0.75-1.70)	1.18 (0.77-1.80)	1.26 (0.82-1.94)	1.38 (0.84-2.24)
2.20 - 4.10	1.68 (1.08-2.62)	1.73 (1.08-2.76)	1.89 (1.19-3.00)	1.97 (1.17-3.31)
4.20 - 212.00	1.99 (1.21-3.27)	2.27 (1.35-3.81)	1.93 (1.15-3.23)	2.30 (1.28-4.13)
P trend	0.003	0.001	0.005	0.003
MHiBP				
0.28 - 0.40	ref	ref	ref	ref
0.50 - 0.80	0.92 (0.61-1.40)	0.96 (0.63-1.48)	0.97 (0.64-1.49)	0.97 (0.60-1.57)
0.90 - 1.60	1.19 (0.76-1.85)	1.23 (0.77-1.96)	0.92 (0.57-1.46)	0.88 (0.52-1.50)
1.70 - 91.70	1.03 (0.63-1.69)	1.21 (0.72-2.03)	0.70 (0.41-1.18)	0.86 (0.48-1.56)
P trend	0.69	0.36	0.20	0.57
ΣDiBP, μmol/L				
0.002 - 0.0057	ref	ref	ref	ref
0.006 - 0.0123	0.81 (0.54-1.24)	0.87 (0.56-1.340	0.86 (0.55-1.34)	0.97 (0.59-1.61)
0.0124 - 0.0247	1.36 (0.87-2.13)	1.44 (0.90-2.29)	1.53 (0.96-2.42)	1.61 (0.95-2.72)
0.0248 - 1.339	1.73 (1.04-2.88)	1.99 (1.16-3.39)	1.49 (0.87-2.53)	1.88 (1.03-3.43)
P trend	0.01	0.004	0.05	0.02
MBzP				
0.40 - 5.90	ref	ref	ref	ref
6.00 - 12.00	1.08 (0.71-1.65)	1.21 (0.78-1.87)	2.19 (1.37-3.50)	2.58 (1.52-4.38)
12.10 - 22.20	0.96 (0.61-1.49)	0.93 (0.59-1.47)	1.67 (1.02-2.74)	1.52 (0.88-2.64)
22.30 - 3590.00	1.45 (0.89-2.37)	1.58 (0.94-2.66)	2.34 (1.37-4.01)	2.73 (1.48-5.04)
P trend	0.21	0.18	0.01	0.01
MCPP				
0.14 - 1.70	ref	ref	ref	ref
1.80 - 3.00	1.18 (0.77-1.81)	1.22 (0.77-1.91)	0.99 (0.62-1.58)	0.98 (0.58-1.67)
3.10 - 5.40	1.40 (0.89-2.20)	1.52 (0.94-2.42)	1.72 (1.08-2.75)	1.86 (1.10-3.16)
5.50 - 108.00	1.64 (0.98-2.74)	1.90 (1.10-3.27)	1.58 (0.93-2.68)	1.78 (0.97-3.28)
P trend	0.05	0.02	0.03	0.02
MEHP				
0.35 - 0.90	ref	ref	ref	ref
1.00 - 1.90	1.29 (0.84-1.96)	1.45 (0.94-2.26)	1.02 (0.65-1.59)	1.03 (0.62-1.71)
2.00 - 4.10	1.45 (0.95-2.22)	1.60 (1.02-2.51)	1.32 (0.85-2.04)	1.46 (0.89-2.42)
4.20 - 367.00	1.43 (0.90-2.27)	1.71 (1.04-2.80)	1.17 (0.73-1.90)	1.31 (0.75-2.27)
P trend	0.09	0.02	0.36	0.20
MEHHP				
0.60 - 9.20	ref	ref	ref	ref
9.30 - 17.10	1.20 (0.79-1.83)	1.26 (0.81-1.95)	1.34 (0.85-2.15)	1.22 (0.72-2.05)
17.20 - 33.00	1.67 (1.09-2.57)	1.80 (1.14-2.83)	2.20 (1.39-3.48)	1.96 (1.17-3.30)
33.20 - 2830.00	2.10 (1.26-3.49)	2.33 (1.36-3.98)	2.97 (1.76-5.03)	2.93 (1.62-5.31)
P trend	0.002	0.001	<0.001	<0.001
MEOHP				
0.20 - 5.80	ref	ref	ref	ref
5.90 - 10.60	1.04 (0.68-1.59)	1.11 (0.71-1.72)	0.99 (0.62-1.58)	0.96 (0.57-1.62)
10.70 - 20.40	1.72 (1.30-2.66)	1.84 (1.16-2.90)	2.05 (1.30-3.24)	1.89 (1.13-3.16)
20.50 - 1610.00	1.80 (1.30-2.98)	2.01 (1.19-3.43)	2.19 (1.30-3.67)	2.40 (1.33-4.32)
P trend	0.006	0.003	<0.001	0.001
MECPP				
1.10 - 12.90	ref	ref	ref	ref
13.00 - 22.60	1.37 (0.91-2.08)	1.45 (0.94-2.23)	1.52 (0.96-2.41)	1.35 (0.80-2.27)
22.70 - 41.50	1.96 (1.26-3.04)	1.98 (1.24-3.16)	2.85 (1.79-4.55)	2.48 (1.46-4.19)
41.60 - 2460.00	2.27 (1.36-3.80)	2.57 (1.49-4.43)	3.15 (1.85-5.37)	3.50 (1.90-6.45)
P trend	0.001	<0.001	<0.001	<0.001
ΣDEHP, μmol/L				
0.008 - 0.1007	ref	ref	ref	ref
0.1008 - 0.1827	1.43 (0.95-2.17)	1.58 (1.03-2.44)	1.28 (0.81-2.03)	1.27 (0.76-2.13)
0.1828 - 0.341	2.22 (1.42-3.47)	2.31 (1.44-3.69)	2.69 (1.69-4.29)	2.33 (1.38-3.94)
0.343 - 24.419	2.27 (1.35-3.82)	2.72 (1.57-4.72)	2.75 (1.61-4.67)	3.29 (1.80-6.03)
P trend	0.001	<0.001	<0.001	<0.001
MCOP				
0.14 - 2.10	ref	ref	ref	ref
2.20 - 3.60	1.78 (1.18-2.67)	2.00 (1.30-3.08)	1.56 (1.00-2.43)	1.50 (0.91-2.49)
3.70 - 6.50	2.10 (1.32-3.34)	2.06 (1.27-3.34)	2.66 (1.66-4.27)	2.38 (1.40-4.05)
6.60 - 239.00	2.79 (1.70-4.57)	2.93 (1.74-4.92)	2.66 (1.59-4.46)	2.55 (1.42-4.58)
P trend	<0.001	<0.001	<0.001	0.001
MCNP				
0.14 - 1.50	ref	ref	ref	ref
1.60 - 2.60	1.43 (0.95-2.16)	1.52 (0.99-2.34)	1.63 (1.04-2.56)	1.63 (0.98-2.72)
2.70 - 4.70	1.34 (0.86-2.11)	1.47 (0.92-2.37)	2.12 (1.33-3.39)	2.37 (1.39-4.04)
4.80 - 91.60	2.00 (1.24-3.22)	2.18 (1.32-3.59)	2.35 (1.42-3.89)	2.64 (1.49-4.67)
P trend	0.006	0.003	0.001	0.001

Abbreviations used: *CI* confidence interval, *MEP* monoethyl phthalate, *MBP* monobutyl phthalate, *MHBP* mono-hydroxybutyl phthalate, *DBP* dibutyl phthalate, *MiBP* mono-isobutyl phthalate, *phthalate MHiBP* mono-hydroxyisobutyl phthalate, *DiBP* di-isobutyl phthalate, *MBzP* monobenzyl phthalate, *MCPP* mono(3-carboxypropyl) phthalate, *MEHP* mono(2-ethylhexyl) phthalate, *MEHHP* mono(2-ethyl-5-hydroxyhexyl) phthalate, *MEOHP* mono(2-ethyl-5-oxohexyl), *DEHP* di(2-ethylhexyl)phthalate, *MECPP* mono(2-ethyl-5-carboxypentyl) phthalate, *MCOP* mono-carboxyoctyl phthalate, *MCNP* mono-carboxynonyl phthalate, *OR* odds ratio

^a^Adjusted for creatinine

^b^Adjusted for creatinine, age, ethnicity, alcohol use, physical activity, smoking status, healthy eating index, dietary energy intake, hormone replacement therapy use, education, income, and history of diabetes, hypertension, dyslipidemia and cardiovascular diseases

Table 4 presents the estimated additional weight change (i.e. beyond the average annual weight change in the study population) associated with phthalate biomarker concentrations after three and 6 years of follow up. We observed positive associations between some phthalate biomarker concentrations and weight change over 3 years (β for 4th vs 1st quartile): MEP (β = 2.32, 95% CI 0.93–3.72; p trend = 0.001), MBP (β = 1.24, 95% CI -0.22-2.50; p trend = 0.05), MHiBP (β = 1.98, 95% CI 0.62–3.33; p trend = 0.02), and MEOHP (β = 1.44, 95% CI 0.07–2.80; p trend = 0.05). These coefficients are interpretable as additional weight gain in the 4th versus 1st quartile of exposure; for example, women in the 4th quartile of MEP gained an additional 2.32 kg over 3 years. Significant associations were observed only in the 4th quartile of each biomarker compared to the 1st quartile. Phthalate biomarker concentrations at baseline were not associated with weight change over 6 years of follow-up (all p trend > 0.10). We did not observe effect modification by baseline BMI for any of the phthalate biomarkers evaluated.

**Table 4:** Estimated additional weight (kg) change associated with phthalate biomarker concentrations, among controls only (*N*=660)

Phthalate Biomarkers, ng/mL	Adjusted^a^ β (95% CI)	
	Year 3	Year 6
MEP		
2.80 - 33.10	ref	ref
33.20 - 67.90	0.07 (-1.35-1.49)	-0.13 (-1.59-1.33)
68.10 - 159.00	-0.28 (-1.71-1.16)	-0.75 (-2.23-0.72)
161.00 - 26000.00	2.32 (0.93-3.72)	1.04 (-0.38-2.47)
P trend	0.001	0.15
MBP		
0.28 - 12.00	ref	ref
12.10 - 23.60	-0.52 (-1.98-0.95)	0.03 (-1.48-1.54)
23.70 - 46.70	0.30 (-1.11-1.72)	0.50 (-0.96-1.96)
46.80 - 3600.00	1.24 (-0.22-2.50)	0.70 (-0.69-2.10)
P trend	0.05	0.26
MHBP		
0.28 - 0.90	ref	ref
1.00 - 1.90	-0.11 (-1.55-1.34)	0.33 (-1.16-1.82)
2.00 - 3.90	0.62 (-0.78-2.02)	-0.02 (-1.45-1.42)
4.00 - 490.00	1.04 (-0.30-2.28)	0.92 (-0.44-2.29)
P trend	0.09	0.23
ΣDBP, μmol/L		
0.002 - 0.065	ref	Ref
0.0652 - 0.132	-0.20 (-1.59-1.19)	-0.20 (-1.63-1.23)
0.133 - 0.264	1.06 (-0.32-2.43)	0.56 (-0.86-1.97)
0.265 - 18.255	0.84 (-0.54-2.22)	0.61 (-0.81-2.03)
P trend	0.11	0.29
MiBP		
0.14 - 1.00	ref	ref
1.10 - 2.10	-0.48 (-1.83-0.87)	0.12 (-1.27-1.51)
2.20 - 4.10	0.49 (-0.88-1.85)	-0.48 (-1.88-0.92)
4.20 - 212.00	-0.27 (-1.63-1.10)	-0.37 (-1.77-1.03)
P trend	0.99	0.48
MHiBP		
0.28 - 0.40	ref	ref
0.50 - 0.80	0.28 (-1.06-1.62)	0.79 (-0.59-2.17)
0.90 - 1.60	-0.34 (-1.71-1.04)	-0.61 (-2.02-0.81)
1.70 - 91.70	1.98 (0.62-3.33)	1.12 (-0.26-2.50)
P trend	0.02	0.33
ΣDiBP, μmol/L		
0.002 - 0.0057	ref	ref
0.006 - 0.0123	0.15 (-1.24-1.54)	1.01 (-0.43-2.44)
0.0124 - 0.0247	-0.10 (-1.46-1.27)	-0.14 (-1.54-1.27)
0.0248 - 1.339	0.95 (-0.44-2.34)	0.15 (-1.28-1.57)
P trend	0.24	0.81
MBzP		
0.40 - 5.90	ref	ref
6.00 - 12.00	-0.14 (-1.55-1.26)	-0.04 (-1.49-1.41)
12.10 - 22.20	-0.44 (-1.87-0.98)	-0.06 (-1.54-1.42)
22.30 - 3590.00	0.94 (-0.47-2.35)	-0.45 (-1.91-1.01)
P trend	0.24	0.54
MCPP		
0.14 - 1.70	ref	Ref
1.80 - 3.00	0.30 (-1.13-1.73)	0.38 (-1.10-1.86)
3.10 - 5.40	-0.65 (-2.07-0.77)	-0.64 (-2.10-0.82)
5.50 - 108.00	0.71 (-0.67-2.10)	-0.13 (-1.55-1.30)
P trend	0.46	0.62
MEHP		
0.35 - 0.90	ref	ref
1.00 - 1.90	0.34 (-1.07-1.75)	-0.15 (-1.60-1.31)
2.00 - 4.10	0.70 (-0.65-2.04)	0.54 (-0.84-1.91)
4.20 - 367.00	-0.24 (-1.66-1.19)	0.41 (-1.66-1.19)
P trend	0.96	0.44
MEHHP		
0.60 - 9.20	ref	ref
9.30 - 17.10	-0.25 (-1.67-1.17)	-0.25 (-1.71-1.21)
17.20 - 33.00	-0.16 (-1.51-1.18)	0.05 (-1.77-1.07)
33.20 - 2830.00	1.06 (-0.32-2.44)	-0.35 (-1.77-1.07)
P trend	0.17	0.73
MEOHP		
0.20 - 5.80	ref	ref
5.90 - 10.60	0.15 (-1.26-1.56)	-0.11 (-1.57-1.36)
10.70 - 20.40	0.23 (-1.13-1.59)	0.21 (-1.18-1.60)
20.50 - 1610.00	1.44 (0.07-2.80)	0.15 (-1.25-1.55)
P trend	0.05	0.76
MECPP		
1.10 - 12.90	ref	ref
13.00 - 22.60	0.58 (-0.81-1.98)	0.88 (-0.55-2.32)
22.70 - 41.50	-0.09 (-1.43-1.25)	0.12 (-1.26-1.50)
41.60 - 2460.00	1.26 (-0.14-2.66)	-0.09 (-1.52-1.35)
P trend	0.17	0.73
ΣDEHP, μmol/L		
0.008 - 0.1007	ref	ref
0.1008 - 0.1827	-0.20 (-1.59-1.18)	-0.30 (-1.73-1.13)
0.1828 - 0.341	-0.01 (-1.37-1.34)	0.07 (-1.32 -1.46)
0.343 - 24.419	1.02 (-0.39-2.43)	-0.41 (-1.87-1.04)
P trend	0.18	0.71
MCOP		
0.14 - 2.10	ref	ref
2.20 - 3.60	0.37 (-0.98-1.72)	0.71 (-0.68-2.11)
3.70 - 6.50	0.23 (-1.20-1.66)	-0.72 (-2.20-0.75)
6.60 - 239.00	-1.17 (-2.54-0.21)	-0.50 (-1.92-0.92)
P trend	0.09	0.25
MCNP		
0.14 - 1.50	ref	ref
1.60 - 2.60	-0.36 (-1.75-1.03)	-0.11 (-1.54-1.33)
2.70 - 4.70	-0.09 (-1.49-1.32)	0.11 (-1.35-1.56)
4.80 - 91.60	-0.39 (-1.77-0.98)	-1.03 (-2.46-0.39)
P trend	0.65	0.17

Abbreviations used: *CI* confidence interval, *MEP* monoethyl phthalate, *MBP* monobutyl phthalate, *MHBP* mono-hydroxybutyl phthalate, *DBP* dibutyl phthalate, *MiBP* mono-isobutyl phthalate, *phthalate MHiBP* mono-hydroxyisobutyl phthalate, *DiBP* di-isobutyl phthalate, *MBzP* monobenzyl phthalate, *MCPP* mono(3-carboxypropyl) phthalate, *MEHP* mono(2-ethylhexyl) phthalate, *MEHHP* mono(2-ethyl-5-hydroxyhexyl) phthalate, *MEOHP* mono(2-ethyl-5-oxohexyl), *DEHP* di(2-ethylhexyl)phthalate, *MECPP* mono(2-ethyl-5-carboxypentyl) phthalate, *MCOP* mono-carboxyoctyl phthalate, *MCNP* mono-carboxynonyl phthalate

^a^Adjusted for creatinine, age, ethnicity, alcohol use, physical activity, smoking status, healthy eating index, dietary energy intake, hormone replacement therapy use, education, income, and history of diabetes, hypertension, dyslipidemia and cardiovascular diseases

## Discussion

In this sample of postmenopausal women from the WHI, we report that concentrations of certain phthalate biomarkers are associated with increased BMI and weight cross-sectionally and also with weight gain over 3 years. Notably, in cross-sectional analyses, women in the highest quartile of ΣDEHP were two times more likely to be overweight (OR 2.72, 95% CI 1.57–4.72) and three times more likely to be obese (OR 3.29, 95% CI 1.80–6.03) compared to those in the lowest quartile. However, in prospective analyses ΣDEHP was not associated with weight gain, and only one DEHP metabolite, MEOHP, was associated: women in the highest quartile of MEOHP concentration gained an additional 1.44 kg over 3 years compared to women with the lowest MEOHP concentrations. Although we observed positive cross-sectional associations between MiBP, MBzP, MCNP, MCOP, MCPP, MECPP, MEHHP, and MEHP and BMI, concentrations of these phthalate biomarkers were not associated with weight change. This suggests that higher concentrations of these phthalate biomarkers might be related to increased exposure to the parent phthalates among the obese, as opposed to a causal relationship with weight gain. Interestingly, concentrations of MEP were inversely associated with obesity in cross-sectional analyses, yet displayed significant positive associations with weight gain over 3 years (4th vs 1st quartile: + 2.32 kg, 95% CI 0.93–3.72; p trend = 0.001). Diethyl phthalate (DEP), the parent compound of MEP, is primarily found in personal care products and cosmetics [14, 15]. One potential explanation of our findings is that use of such products varies by BMI category, thus confounding the cross-sectional associations between MEP and BMI, yet exposure to DEP via these products may promote weight gain. Future studies will be useful in elucidating the potential effect of DEP exposure on body weight.

The few prior cross-sectional evaluations of urinary phthalate metabolites and BMI in adult women produced inconsistent results. Overall, our findings are generally consistent with those of Buser et al [4] (using 2007–2010 NHANES data) but are in contrast with the other studies. Specifically, we and Buser et al [4] reported positive associations with MECPP, MEHHP, MEOHP, and DEHP, while Yaghjyan et al [5] (using 1999–2004 NHANES data) reported inverse associations. Yaghjyan et al [5] also reported a positive association with MEHP, which agrees with our findings; however, an early study by Hatch et al [6] (using 1999–2002 NHANES data) reported an inverse association between MEHP and BMI and Buser et al [4] observed no association. Our findings support the positive association between MCOP and BMI reported by Buser et al [4], although they reported no association with MCNP while we observed a positive association. The inverse association we observed with MEP contrasts with the non-significant positive trends reported by Hatch et al [6] and Buser et al [4], although no association was observed by Yaghjyan et al [5] or Song et al [7] (using a sub-sample of NHS/NHS2 participants). We report no significant association between MBP and BMI, in agreement with Buser et al [4], while an inverse association was observed by Hatch et al [6] and Song et al [7], and Yaghjyan et al [5] observed a positive association. Finally, the positive association we observed between MBzP and BMI is in contrast to the inverse association reported by Song et al [7] and the null findings of Hatch et al [6], Buser et al [4], and Yaghjyan et al [5].

The reasons for these differences across studies are unclear, but may relate to differences in population exposure to phthalates over time. This is unlikely to fully explain observed differences, however, given that our results most closely align with the Buser et al [4] study, which used samples donated more than a decade following ours. Additionally, the DEHP metabolites measured in NHANES varied over time, with only MEHP measured in 1999–2000, MEOHP and MEHHP first measured in 2001–2002, and MECPP first measured in 2003–2004. Also, differences in the demographic characteristics of our highly selected population compared to those of NHANES participants, which are randomly selected to be representative of the general U.S. population, and the older age of our participants as compared to NHS/NHS2 participants may have affected results. Another important factor contributing to the inconsistency across studies may be the relatively high within-person variability in concentrations of several urinary phthalate metabolites [16], which may result in substantial non-differential misclassification. In sum, our results and those of Buser et al [4] support positive cross-sectional associations between some phthalate metabolites, especially the DEHP metabolites, and BMI category.

Importantly, the observed positive prospective associations between some phthalate biomarkers and weight change over 3 years did not persist over 6 years. Since the half-lives of phthalate metabolites are short (6–12 h) and urinary concentrations exhibit within-person variability over time [16], a single measurement of phthalate metabolites taken at baseline is unlikely to fully characterize an individual’s long-term exposure. Thus, a single measurement of a given phthalate exposure biomarker may predict weight gain over short time periods, but not over longer time periods.

Only one prior study prospectively evaluated associations between phthalate exposure and weight change. In a sample of NHS and NHS2 participants, Song and colleagues [7] reported significantly higher annual weight gain associated with MBzP (β = 0.42; 95% CI: 0.26–0.57, p-trend< 0.001), phthalic acid (a non-specific biomarker of phthalate exposure) (β = 0.33; 95% CI: 0.15–0.50, p-trend = 0.001), the sum of butyl phthalate metabolites (MiBP and MBP) (β = 0.34; 95% CI: 0.18–0.50, p-trend < 0.001) and the total sum of all the phthalate metabolites measured (β = 0.17; 95% CI: 0.02–0.33, p-trend = 0.05). Our observed 1.24 kg increased weight gain over 3 years among associated with the highest quartile of MBP is consistent with their reported 0.34 kg annual weight gain, which would be equivalent to a gain of 1.02 kg over 3 years. We did not observe statistically significantly increased weight gain associated with MBzP, however. We also observed increased weight gain associated with higher concentrations of MEOHP, MEP, and MHiBP; MEP was not associated with weight gain in the Song et al [7] study, and MEOHP and MHiBP were not separately evaluated. Our study population was, on average, older and of higher baseline weight than the NHS/NHS2 study population, which could explain some differences between our findings, given that patterns of weight gain and loss vary among older age groups. Also, Song et al [7] utilized self-reported weight over a 10-year period as their outcome, while we utilized measured weight over 3- and 6-year intervals. Future work will benefit from utilizing repeated measurements of phthalate biomarkers over extended time periods.

It is noteworthy that we observed some inconsistent associations between the cross-sectional and the prospective analyses. Specifically, we observed that MEP was inversely associated with BMI in the cross-sectional analysis, yet positively associated with weight gain in the prospective analysis. Also, MCNP, MCOP, and MECPP, were positively associated with BMI in the cross-sectional analysis yet were not associated with prospective weight gain. These differences may be explained in part by differences in the study population used for the cross-sectional and prospective analyses; we excluded women later diagnosed with breast cancer from the prospective analysis, which resulted in a significantly reduced sample size (997 vs 660) and loss of statistical power. Cases and controls also may differ in ways that could have affected results, although we observed similar cross-sectional results among controls. Another potential explanation is that phthalate biomarker concentrations may reflect exposure to a source of phthalates (e.g. processed foods) that is also associated with obesity, and thus the cross-sectional associations we observed could be due to reverse causality. Overweight/obese woman may have demographic characteristics (e.g. non-White, lower socioeconomic status) and/or engage in health behaviors (e.g. alcohol use, poor diet quality, consumption of canned/processed foods) that are associated with higher phthalate exposure. Additionally, overweight and obese women are more likely to have comorbidities that result in more frequent contact with medical equipment and medications, which also may cause increased exposure to certain phthalates. While we adjusted for many factors including race/ethnicity, dietary quality and energy intake, and comorbidities, it is possible that residual confounding affected our results.

Laboratory evidence suggests several mechanisms by which phthalates could act as obesogens. Some phthalates may induce adipogenesis by activating metabolic sensors, such as the peroxisome proliferator-activated receptors (PPARs) [2]. PPARs are transcription factors that play an essential role in energy metabolism. PPARγ, expressed in the endothelial, vascular and smooth muscle cells, controls fatty acids storage in the adipose tissue by promoting adipocyte differentiation [17]. Evidence from experimental studies has shown that MBzP, mono-sec-butyl phthalate, and MEHP [2, 3], directly activate PPARγ and stimulate a dose dependent increase in adipocyte differentiation [3], which in turn promotes adipogenesis. Furthermore, MEP has been shown to increase activity of human PPARγ [18]. Phthalates also are known to disrupt the thyroid hormone system, which also could affect weight gain. Triiodothyronine (T3), a thyroid hormone, regulates adipogenesis by controlling genes that are involved in lipolysis and lipogenesis [19, 20]. In a recent study DBP and DEHP were shown to inhibit the regular activity of T3, with DBP acting as a stronger antagonist than DEHP [21]. Such findings are consistent with our reported positive associations between some DBP and DEHP metabolites (MBP, MEOHP) and weight gain.

Our study has several important strengths. We utilized objectively measured data on height and weight, which reduces misclassification in the outcome measures. We also considered a broad range of variables as potential confounders using the extensive WHI data resource. We considered a broad panel of 13 phthalate metabolites, which allowed for a more complete evaluation of phthalate exposure in our population than in prior studies.

Our findings should be interpreted in light of additional relevant limitations. We used a single measure of urinary phthalate biomarkers to estimate the study participants’ long-term exposure to phthalates. Phthalates are rapidly metabolized and excreted within hours, and a single measurement may not accurately reflect the long-term exposure levels of our participants. Furthermore, the within person variability of the phthalate metabolites could lead to non-differential misclassification of the exposure and attenuate the true associations; therefore, we may have failed to detect some true associations and others may be even stronger than we have reported. Additionally, urinary creatinine is an imperfect correction for urine dilution and could be associated with BMI and/or factors predictive of BMI [22]. Type I error also is possible given the number of statistical tests performed. Also, under-reporting of energy intake by overweight/obese participants has been reported in WHI [23, 24], which could affect the HEI-2005 scores and cause residual confounding, though we expect this to have minimal effects on our estimates because diet is not the major source of phthalate exposure. Some participants were excluded from the full analyses because of missing data on covariates. While multiple imputation is useful when missing data are present, we were concerned that the resulting increased confidence interval width would further obscure any potential associations given the measurement error that already affected our analyses. In sensitivity analyses of the cross-sectional study which included 1187 participants instead of the 997 reported in the Results section, results were generally similar for all phthalate biomarkers, thus we anticipate that any potential bias from our complete case analysis was minimal. Lastly, we utilized a highly selected population of postmenopausal women, with limited racial/ethnic diversity. Future work in populations including men, younger women, and increased minority representation will be useful to fully understand whether phthalates can affect weight gain in other populations.

## Conclusions

We provide evidence that certain phthalates may contribute to weight gain among postmenopausal women at 3 years of follow-up, but not at 6 years of follow-up. Additional research to characterize the potential role of phthalates in development of obesity is important given the continued obesity epidemic and the ubiquity of these chemicals.

## Additional file

Additional file 1: **Table S1.** Cross-sectional associations between phthalate biomarker concentrations and weight within the WHI (*N* = 997). **Table S2.** Cross-sectional associations between phthalate biomarker concentrations and overweight and obesity compared to underweight/normal within the WHI, among controls only (*N* = 660). **Table S3.** Cross-sectional associations between phthalate biomarker concentrations and weight within the WHI, among controls only (*N* = 660). **Table S4.** Cross-sectional associations between phthalate biomarker concentrations and overweight and obesity compared to underweight/normal within the WHI, among participants with complete data on a reduced set of covariates (*N* = 1187). (DOCX 54 kb)
