# Supplementary material for: Urinary concentrations of phthalate biomarkers and weight change among postmenopausal women: a prospective cohort study
Source: Environ Health. 2019 Mar 12;18:20. doi: 10.1186/s12940-019-0458-6 (PMC6417117; doi:10.1186/s12940-019-0458-6)
Supplement: Supplementary file 1 — Table S1. Cross-sectional associations between phthalate biomarker concentrations and weight within the WHI (N = 997). Table S2. Cross-sectional associations between phthalate biomarker concentrations and overweight and obesity compared to underweight/normal within the WHI, among controls only (N = 660). Table S3. Cross-sectional associations between phthalate biomarker concentrations and weight within the WHI, among controls only (N = 660). Table S4. Cross-sectional associations between phthalate biomarker concentrations and overweight and obesity compared to underweight/normal within the WHI, among participants with complete data on a reduced set of covariates (N = 1187). (DOCX 54 kb) [file 12940_2019_458_MOESM1_ESM.docx]

**Additional file 1:**

**Table S1: Cross-sectional associations between phthalate biomarker concentrations and weight within the WHI (N=997).**

| Phthalate Biomarker, ng/mL | β (95% CI) | |
| --- | --- | --- |
|  | Model 1^a^ | Model 2^b^ |
| MEP |  |  |
| 2.80 - 33.10 | ref | ref |
| 33.20 - 67.90 | -1.82 (-4.59-0.95) | -2.32 (-4.84-0.20) |
| 68.10 - 159.00 | -2.59 (5-.44-0.25) | -3.45 (-6.03-^-^0.86) |
| 161.00 - 26000.00 | -2.65 (-5.55-0.24) | -3.40 (-6.04-^-^0.75) |
| P trend | 0.08 | 0.02 |
| MBP |  |  |
| 0.28 - 12.00 | ref | ref |
| 12.10 - 23.60 | 1.06 (-1.77-3.89) | 1.24 (-1.34-3.81) |
| 23.70 - 46.70 | 0.12 (-2.74-2.99) | -0.09 (-2.70-2.52) |
| 46.80 - 3600.00 | -3.11 (-6.19-^-^0.04) | -2.42 (-5.26-0.41) |
| P trend | 0.04 | 0.07 |
| MHBP |  |  |
| 0.28 - 0.90 | ref | ref |
| 1.00 - 1.90 | -1.51 (-4.26-1.23) | -1.36 (-3.86-1.14) |
| 2.00 - 3.90 | -2.19 (-5.01-0.63) | -1.77 (-4.35-0.82) |
| 4.00 - 490.00 | -4.09 (-7.24-0.93) | -5.34 (-8.07-^-^2.61) |
| P trend | <0.001 | <0.001 |
| ΣDBP, μmol/L |  |  |
| 0.002 - 0.065 | ref | ref |
| 0.0652 - 0.132 | 0.37 (-2.31-3.05) | 0.19 (-2.25-2.63) |
| 0.133 - 0.264 | -1.02 (-3.83-1.79) | -0.95 (-3.52-1.61) |
| 0.265 - 18.255 | -3.35 (-6.47-^-^0.21) | -2.45 (-5.32-0.42) |
| P trend | 0.04 | 0.09 |
| MiBP |  |  |
| 0.14 - 1.00 | ref | ref |
| 1.10 - 2.10 | 0.93 (-1.73-3.59) | 0.62 (-1.80-3.05) |
| 2.20 - 4.10 | 2.06 (-0.74-4.86) | 1.15 (-1.40-3.70) |
| 4.20 - 212.00 | 1.09 (-2.02-4.20) | 0.94 (-1.90-3.78) |
| P trend | 0.34 | 0.44 |
| MHiBP |  |  |
| 0.28 - 0.40 | ref | ref |
| 0.50 - 0.80 | -0.006 (-2.63-2.62) | -0.40 (-2.80-1.99) |
| 0.90 - 1.60 | -1.69 (-4.51-1.12) | -1.98 (-4.55-0.60) |
| 1.70 - 91.70 | -4.09 (-7.24-^-^0.93) | -3.22 (-6.11-^-^0.33) |
| P trend | 0.01 | 0.02 |
| ΣDiBP, μmol/L |  |  |
| 0.002 - 0.0057 | ref | ref |
| 0.006 - 0.0123 | -1.98 (-4.72-0.75) | -1.61 (-4.11-0.88) |
| 0.0124 - 0.0247 | 1.11 (-1.70-3.92) | 0.54 (-2.02-3.10) |
| 0.0248 - 1.339 | -1.32 (-4.51-1.87) | -0.85 (-3.76-2.06) |
| P trend | 0.82 | 0.90 |
| MBzP |  |  |
| 0.40 - 5.90 | ref | ref |
| 6.00 - 12.00 | 2.07 (-0.70-4.83) | 2.09 (-0.10-4.89) |
| 12.10 - 22.20 | 1.12 (-1.79-4.03) | 0.04 (-2.59-2.68) |
| 22.30 - 3590.00 | 0.11 (-3.04-3.25) | -0.13 (-3.01-2.74) |
| P trend | 0.94 | 0.65 |
| MCPP |  |  |
| 0.14 - 1.70 | ref | ref |
| 1.80 - 3.00 | -2.05 (-4.88-0.78) | -1.81 (-4.40-0.78) |
| 3.10 - 5.40 | 0.40 (-2.46-3.26) | -0.25 (-2.87-2.36) |
| 5.50 - 108.00 | -0.28 (-3.52-2.96) | -0.33 (-3.30-2.65) |
| P trend | 0.81 | 0.94 |
| MEHP |  |  |
| 0.35 - 0.90 | Ref | Ref |
| 1.00 - 1.90 | 0.17 (-2.57-2.92) | 0.31 (-2.19-2.81) |
| 2.00 - 4.10 | -0.84 (-3.55-1.87) | -1.32 (-3.80-1.16) |
| 4.20 - 367.00 | -0.60 (-3.56-2.35) | -0.50 (-3.22-2.21) |
| P trend | 0.58 | 0.51 |
| MEHHP |  |  |
| 0.60 - 9.20 | ref | ref |
| 9.30 - 17.10 | 1.76 (-1.03-4.56) | 0.96 (-1.58-3.52) |
| 17.20 - 33.00 | 2.81 (0.06-5.56) | 1.05 (-1.49-3.58) |
| 33.20 - 2830.00 | 3.27 (0.13-6.41) | 2.12 (-0.75-5.00) |
| P trend | 0.03 | 0.16 |
| MEOHP |  |  |
| 0.20 - 5.80 | ref | ref |
| 5.90 - 10.60 | -0.31 (-3.11-2.49) | -0.59 (-3.14-1.96) |
| 10.70 - 20.40 | 2.07 (-0.68-4.82) | 0.73 (-1.79-3.25) |
| 20.50 - 1610.00 | 2.41 (-0.73-5.54) | 2.09 (-0.77-4.96) |
| P trend | 0.07 | 0.13 |
| MECPP |  |  |
| 1.10 - 12.90 | ref | ref |
| 13.00 - 22.60 | 2.14 (-0.61-4.89) | 0.82 (-1.68-3.33) |
| 22.70 - 41.50 | 3.87 (1.09-6.65) | 1.72 (-0.84-4.29) |
| 41.60 - 2460.00 | 3.16 (-0.01-6.34) | 2.76 (-0.17-5.69) |
| P trend | 0.02 | 0.05 |
| ΣDEHP, μmol/L |  |  |
| 0.008 - 0.1007 | ref | ref |
| 0.1008 - 0.1827 | 1.26 (-1.48-4.00) | 0.72 (-1.78-3.22) |
| 0.1828 - 0.341 | 3.77 (0.98-6.55) | 1.65 (-0.92-4.22) |
| 0.343 - 24.419 | 2.31 (-0.90-5.52) | 2.21 (-0.73-5.15) |
| P trend | 0.06 | 0.11 |
| MCOP |  |  |
| 0.14 - 2.10 | ref | ref |
| 2.20 - 3.60 | 1.08 (-1.60-3.76) | 0.36 (-2.09-2.81) |
| 3.70 - 6.50 | 3.80 (0.92-6.67) | 2.32 (-0.30-4.94) |
| 6.60 - 239.00 | 3.82 (0.75-6.88) | 3.00 (0.17-5.83) |
| P trend | 0.007 | 0.03 |
| MCNP |  |  |
| 0.14 - 1.50 | ref | ref |
| 1.60 - 2.60 | 2.03 (-0.67-4.73) | 1.67 (-0.80-4.14) |
| 2.70 - 4.70 | 3.10 (0.24-5.96) | 2.79 (0.18-5.41) |
| 4.80 - 91.60 | 2.65 (-0.36-5.66) | 2.40 (-0.34-5.15) |
| P trend | 0.08 | 0.08 |
| ^a^Adjusted for creatinine | | |
| ^b^Adjusted for creatinine, age, ethnicity, alcohol use, physical activity, smoking status, healthy eating index, dietary energy intake, hormone replacement therapy use, education, income, and history of diabetes, hypertension, dyslipidemia and cardiovascular diseases | | |
| Abbreviations used: MEP, monoethyl phthalate; MBP, monobutyl phthalate; MHBP, mono-hydroxybutyl phthalate; DBP, dibutyl phthalate; MiBP, mono-isobutyl phthalate; phthalate MHiBP, mono-hydroxyisobutyl phthalate; DiBP, di-isobutyl phthalate; MBzP, monobenzyl phthalate; MCPP, mono(3-carboxypropyl) phthalate; MEHP, mono(2-ethylhexyl) phthalate; MEHHP, mono(2-ethyl-5-hydroxyhexyl) phthalate; MEOHP, mono(2-ethyl-5-oxohexyl); DEHP, di(2-ethylhexyl)phthalate; MECPP, mono(2-ethyl-5-carboxypentyl) phthalate; MCOP, mono-carboxyoctyl phthalate; MCNP, mono-carboxynonyl phthalate | | |

**Table S2: Cross-sectional associations between phthalate biomarker concentrations and overweight and obesity compared to underweight/normal within the WHI, among controls only (N=660).**

| **Phthalate Biomarkers, ng/mL** |  |  |  |  |
| --- | --- | --- | --- | --- |
|  | **Overweight**  **OR (95% CI)** | | **Obese**  **OR (95% CI)** | |
|  | **Model 1^a^** | **Model 2^b^** | **Model 1^a^** | **Model 2^b^** |
| MEP |  |  |  |  |
| 2.80 - 33.10 | ref | ref | ref | ref |
| 33.20 - 67.90 | 0.85 (0.51-1.44) | 0.86 (0.50-1.50) | 0.82 (0.46-1.47) | 0.64 (0.33-1.24) |
| 68.10 - 159.00 | 0.76 (0.44-1.31) | 0.72 (0.4-1.27) | 0.92 (0.51-1.66) | 0.67 (0.34-1.31) |
| 161.00 - 26000.00 | 0.51 (0.29-0.90) | 0.53 (0.29-0.96) | 0.72 (0.40-1.31) | 0.56 (0.28-1.11) |
| P trend | 0.02 | 0.03 | 0.33 | 0.15 |
| MBP |  |  |  |  |
| 0.28 - 12.00 | ref | ref | ref | ref |
| 12.10 - 23.60 | 1.07 (0.63-1.80) | 1.05 (0.60-1.81) | 1.16 (0.63-2.12) | 1.12 (0.56-2.23) |
| 23.70 - 46.70 | 0.98 (0.57-1.68) | 1.03 (0.59-1.82) | 1.68 (0.94-3.02) | 2.09 (1.07-4.06) |
| 46.80 - 3600.00 | 0.95 (0.54-1.68) | 1.06 (0.58-1.94) | 1.35 (0.73-2.53) | 1.75 (0.86-3.55) |
| P trend | 0.81 | 0.87 | 0.24 | 0.06 |
| MHBP |  |  |  |  |
| 0.28 - 0.90 | ref | ref | ref | ref |
| 1.00 - 1.90 | 0.91 (0.54-1.54) | 0.84 (0.48-1.45) | 0.88 (0.49-1.60) | 1.01 (0.52-1.99) |
| 2.00 - 3.90 | 0.85 (0.50-1.48) | 0.95 (0.53-1.69) | 1.47 (0.83-2.61) | 2.10 (1.08-4.06) |
| 4.00 - 490.00 | 0.57 (0.33-0.99) | 0.59 (0.33-1.07) | 0.61 (0.33-1.12) | 0.88 (0.43-1.77) |
| P trend | 0.05 | 0.11 | 0.25 | 0.92 |
| ΣDBP, μmol/L |  |  |  |  |
| 0.002 - 0.065 | ref | ref | ref | ref |
| 0.0652 - 0.132 | 0.98 (0.60-1.62) | 0.99 (0.58-1.67) | 1.56 (0.88-2.75) | 1.49 (0.78-2.85) |
| 0.133 - 0.264 | 0.92 (0.54-1.55) | 1.01 (0.58-1.76) | 1.64 (0.92-2.92) | 2.30 (1.19-4.43) |
| 0.265 - 18.255 | 0.90 (0.50-1.60) | .0.98 (0.53-1.81) | 1.42 (0.75-2.69) | 1.86 (0.90-3.81) |
| P trend | 0.68 | 0.96 | 0.26 | 0.05 |
| MiBP |  |  |  |  |
| 0.14 - 1.00 | ref | ref | ref | ref |
| 1.10 - 2.10 | 1.34 (0.82-2.20) | 1.28 (0.7-2.16) | 1.49 (0.86-2.57) | 1.58 (0.84-2.95) |
| 2.20 - 4.10 | 1.44 (0.85-2.45) | 1.42 (0.81-2.47) | 1.68 (0.95-2.98) | 1.99 (0.93-1.00) |
| 4.20 - 212.00 | 1.80 (1.00-3.26) | 2.01 (1.09-3.72) | 2.33 (1.25-4.35) | 2.97 (1.46-6.05) |
| P trend | 0.05 | 0.03 | 0.008 | 0.002 |
| MHiBP | |  |  |  |
| 0.28 - 0.40 | ref | ref | ref | ref |
| 0.50 - 0.80 | 0.92 (0.56-1.51) | 0.98 (0.59-1.66) | 0.92 (0.54-1.57) | 1.09 (0.60-2.00) |
| 0.90 - 1.60 | 1.11 (0.65-1.90) | 1.13 (0.64-1.98) | 0.98 (0.55-1.74) | 1.01 (0.53-1.92) |
| 1.70 - 91.70 | 1.08 (0.60-1.94) | 1.30 (0.70-2.40) | 0.86 (0.46-1.61) | 1.18 (0.58-2.41) |
| P trend | 0.71 | 0.39 | 0.68 | 0.71 |
| ΣDiBP, μmol/L |  |  |  |  |
| 0.002 - 0.0057 | ref | ref | ref | ref |
| 0.006 - 0.0123 | 0.87 (0.53-1.45) | 0.84 (0.49-1.42) | 1.07 (0.61-1.87) | 1.17 (0.62-2.22) |
| 0.0124 - 0.0247 | 1.27 (0.75-2.15) | 1.27 (0.72-2.21) | 1.47 (0.83-2.62) | 1.75 (0.90-3.38) |
| 0.0248 - 1.339 | 1.58 (0.857-2.87) | 1.78 (0.95-3.33) | 1.89 (1.00-3.57) | 2.57 (1.24-5.32) |
| P trend | 0.09 | 0.05 | 0.04 | 0.008 |
| MBzP |  |  |  |  |
| 0.40 - 5.90 | ref | ref | ref | ref |
| 6.00 - 12.00 | 0.96 (0.57-0.61) | 0.97 (0.56-1.68) | 3.14 (1.70-5.801) | 3.60 (1.80-7.20) |
| 12.10 - 22.20 | 0.84 (0.50-1.40) | 1.00 (0.99-1.00) | 1.71 (0.90-3.24) | 1.46 (0.72-2.99) |
| 22.30 - 3590.00 | 1.24 (0.69-2.22) | 1.27 (0.68-2.37) | 2.93 (1.48-5.79) | 3.86 (1.77-8.45) |
| P trend | 0.64 | 0.75 | 0.02 | 0.02 |
| MCPP | |  |  |  |
| 0.14 - 1.70 | ref | ref | ref | ref |
| 1.80 - 3.00 | 1.15 (0.69-1.92) | 1.20 (0.70-2.06) | 1.03 (0.56-1.87) | 1.04 (0.53-2.06) |
| 3.10 - 5.40 | 1.39 (0.80-2.43) | 1.39 (0.77-2.49) | 2.39 (1.32-4.31) | 2.30 (1.18-4.49) |
| 5.50 - 108.00 | 1.72 (0.93-3.16) | 1.86 (0.97-3.56) | 1.88 (0.97-3.64) | 2.10 (0.98-4.51) |
| P trend | 0.07 | 0.06 | 0.02 | 0.02 |
| MEHP |  |  |  |  |
| 0.35 - 0.90 | ref | ref | ref | ref |
| 1.00 - 1.90 | 1.36 (0.82-2.28) | 1.44 (0.84-2.47) | 1.06 (0.60-1.87) | 1.03 (0.54-1.96) |
| 2.00 - 4.10 | 1.43 (0.85-2.40) | 1.50 (0.86-2.60) | 1.64 (0.95-2.82) | 1.68 (0.90-3.14) |
| 4.20 - 367.00 | 1.49 (0.85-2.61) | 1.75 (0.96-3.18) | 1.12 (0.61-2.05) | 1.26 (0.62-2.54) |
| P trend | 0.14 | 0.06 | 0.40 | 0.29 |
| MEHHP | |  |  |  |
| 0.60 - 9.20 | ref | ref | ref | ref |
| 9.30 - 17.10 | 1.02 (0.62-1.69) | 1.03 (0.60-1.76) | 1.08 (0.60-1.94) | 0.95 (0.49-1.84) |
| 17.20 - 33.00 | 1.75 (1.05-2.94) | 1.84 (1.06-3.20) | 2.46 (1.40-4.34) | 2.13 (1.12-4.06) |
| 33.20 - 2830.00 | 1.97 (1.07-3.62) | 2.04 (1.07-3.90) | 2.95 (1.54-5.64) | 2.70 (1.29-5.63) |
| P trend | 0.01 | 0.01 | <0.001 | 0.002 |
| MEOHP | |  |  |  |
| 0.20 - 5.80 | ref | ref | ref | ref |
| 5.90 - 10.60 | 1.09 (0.66-1.81) | 1.12 (0.66-1.91) | 0.86 (0.48-1.55) | 0.80 (0.41-1.55) |
| 10.70 - 20.40 | 1.73 (1.02-2.93) | 1.78 (1.01-3.11) | 2.12 (1.20-3.73) | 1.92 (1.01-3.63) |
| 20.50 - 1610.00 | 1.62 (0.89-2.95) | 1.69 (0.89-3.21) | 2.10 (0.89-2.95) | 1.96 (0.95-4.03) |
| P trend | 0.05 | 0.05 | 0.004 | 0.02 |
|  |  |  |  |  |
| MECPP | |  |  |  |
| 1.10 - 12.90 | ref | ref | ref | ref |
| 13.00 - 22.60 | 1.32 (0.80-2.18) | 1.30 (0.77-2.21) | 1.18 (0.66-2.13) | 1.01 (0.52-1.95) |
| 22.70 - 41.50 | 2.10 (1.24-3.56) | 2.07 (1.18-3.65) | 2.38 (1.27-4.45) | 2.47 (1.28-4.73) |
| 41.60 - 2460.00 | 2.38 (1.27-4.45) | 2.55 (1.29-5.02) | 3.31 (1.71-6.41) | 3.39 (1.57-7.31) |
| P trend | 0.002 | 0.003 | <0.001 | <0.001 |
| ΣDEHP, μmol/L |  |  |  |  |
| 0.008 - 0.1007 | ref | ref | ref | ref |
| 0.1008 - 0.1827 | 1.44 (0.87-2.36) | 1.54 (0.91-2.62) | 1.09 (0.61-1.95) | 1.08 (0.56-2.10) |
| 0.1828 - 0.341 | 2.32 (1.36-3.97) | 2.35 (1.32-4.19) | 2.87 (1.62-5.09) | 2.41 (1.25-4.64) |
| 0.343 - 24.419 | 2.30 (1.23-4.30) | 2.73 (1.39-5.38) | 2.83 (1.47-5.45) | 3.33 (1.56-7.14) |
| P trend | 0.002 | 0.001 | <0.001 | <0.001 |
| MCOP |  |  |  |  |
| 0.14 - 2.10 | ref | ref | ref | ref |
| 2.20 - 3.60 | 1.49 (0.91-2.44) | 1.60 (0.95-2.69) | 1.14 (0.66-1.98) | 1.25 (0.67-2.33) |
| 3.70 - 6.50 | 2.16 (1.21-3.85) | 2.08 (1.14-3.81) | 2.43 (1.33-4.34) | 2.53 (1.28-4.99) |
| 6.60 - 239.00 | 2.75 (1.51-5.00) | 2.69 (1.43-5.06) | 2.27 (1.21-4.29) | 2.20 (1.05-4.57) |
| P trend | 0.001 | 0.002 | 0.003 | 0.01 |
| MCNP |  |  |  |  |
| 0.14 - 1.50 | ref | ref | ref | ref |
| 1.60 - 2.60 | 1.25 (0.75-2.07) | 1.19 (0.70-2.02) | 1.44 (0.82-2.54) | 1.37 (0.72-2.60) |
| 2.70 - 4.70 | 1.32 (0.97-3.11) | 1.28 (0.72-2.26) | 1.73 (0.97-3.11) | 1.75 (0.90-3.43) |
| 4.80 - 91.60 | 2.01 (1.14-3.56) | 2.01 (1.10-3.66) | 2.16 (1.16-4.03) | 2.47 (1.22-5.00) |
| P trend | 0.02 | 0.02 | 0.02 | 0.01 |
| ^a^Adjusted for creatinine  ^b^Adjusted for creatinine, age, ethnicity, alcohol use, physical activity, smoking status, healthy eating index, dietary energy intake, hormone replacement therapy use, education, income, and history of diabetes, hypertension, dyslipidemia and cardiovascular diseases | | | | |
| Abbreviations used: MEP, monoethyl phthalate; MBP, monobutyl phthalate; MHBP, mono-hydroxybutyl phthalate; DBP, dibutyl phthalate; MiBP, mono-isobutyl phthalate; phthalate MHiBP, mono-hydroxyisobutyl phthalate; DiBP, di-isobutyl phthalate; MBzP, monobenzyl phthalate; MCPP, mono(3-carboxypropyl) phthalate; MEHP, mono(2-ethylhexyl) phthalate; MEHHP, mono(2-ethyl-5-hydroxyhexyl) phthalate; MEOHP, mono(2-ethyl-5-oxohexyl); DEHP, di(2-ethylhexyl)phthalate; MECPP, mono(2-ethyl-5-carboxypentyl) phthalate; MCOP, mono-carboxyoctyl phthalate; MCNP, mono-carboxynonyl phthalate | | | | |

**Table S3: Cross-sectional associations between phthalate biomarker concentrations and weight within the WHI, among controls only (N=660).**

| Phthalate Biomarker, ng/mL | β (95% CI) | |
| --- | --- | --- |
|  | Model 1^a^ | Model 2^b^ |
| MEP |  |  |
| 2.80 - 33.10 | ref | ref |
| 33.20 - 67.90 | -1.49 (-4.86-1.88) | -2.72 (-5.81-0.37) |
| 68.10 - 159.00 | -1.66 (-5.14-1.82) | -3.23 (-6.43-0.04) |
| 161.00 - 26000.00 | -1.92 (-5.49-1.65) | -2.81 (-6.12-0.50) |
| P trend | 0.33 | 0.15 |
| MBP |  |  |
| 0.28 - 12.00 | ref | ref |
| 12.10 - 23.60 | 1.35 (-2.09-4.80) | 0.53 (-2.63-3.69) |
| 23.70 - 46.70 | 1.34 (-2.10-4.79) | 1.45 (-1.70-4.60) |
| 46.80 - 3600.00 | -1.40 (-5.07-2.27) | -1.16 (-4.54-2.23) |
| P trend | 0.48 | 0.62 |
| MHBP |  |  |
| 0.28 - 0.90 | ref | ref |
| 1.00 - 1.90 | -1.49 (-4.85-1.88) | -1.48 (-4.57-1.61) |
| 2.00 - 3.90 | 0.40 (-2.97-3.77) | 0.86 (-2.24-3.96) |
| 4.00 - 490.00 | -6.17 (-9.70-^-^2.63) | -4.50 (-7.80-^-^1.21) |
| P trend | 0.003 | 0.03 |
| ΣDBP, μmol/L |  |  |
| 0.002 - 0.065 | ref | ref |
| 0.0652 - 0.132 | 1.30 (-1.97-4.58) | 0.36 (-2.64-3.35) |
| 0.133 - 0.264 | 0.57 (-2.81-3.94) | 1.41 (-1.68-4.50) |
| 0.265 - 18.255 | -1.90 (-5.63-1.83) | -1.22 (-4.66-2.22) |
| P trend | 0.37 | 0.70 |
| MiBP |  |  |
| 0.14 - 1.00 | ref | ref |
| 1.10 - 2.10 | 1.20 (-2.01-4.42) | 0.63 (-2.32-3.58) |
| 2.20 - 4.10 | 0.99 (-2.41-4.38) | 0.87 (-2.23-3.97) |
| 4.20 - 212.00 | 1.99 (-1.71-5.69) | 1.76 (-1.58-5.10) |
| P trend | 0.31 | 0.31 |
| MHiBP |  |  |
| 0.28 - 0.40 | ref | ref |
| 0.50 - 0.80 | -1.59 (-4.79-1.61) | -0.88 (-3.79-2.03) |
| 0.90 - 1.60 | -1.98 (-5.40-1.43) | -2.18 (-5.29-0.93) |
| 1.70 - 91.70 | -2.99 (-6.72-0.74) | -1.61 (-5.01-1.79) |
| P trend | 0.11 | 0.26 |
| ΣDiBP, μmol/L |  |  |
| 0.002 - 0.0057 | ref | ref |
| 0.006 - 0.0123 | -1.75 (-5.07-1.57) | -1.89 (-4.92-1.14) |
| 0.0124 - 0.0247 | 0.19 (-3.21-3.58) | 0.22 (-2.89-3.32) |
| 0.0248 - 1.339 | -0.29 (-4.05-3.46) | 0.09 (-3.32-3.49) |
| P trend | 0.92 | 0.73 |
| MBzP |  |  |
| 0.40 - 5.90 | ref | ref |
| 6.00 - 12.00 | 3.27 (-0.09-6.62) | 2.71 (-0.34-5.77) |
| 12.10 - 22.20 | -0.02 (-3.48-3.44) | -1.42 (-4.55-1.71) |
| 22.30 - 3590.00 | 0.07 (-3.69-3.82) | 0.11 (-3.35-3.56) |
| P trend | 0.71 | 0.55 |
| MCPP |  |  |
| 0.14 - 1.70 | ref | ref |
| 1.80 - 3.00 | -1.59 (-5.00-1.81) | -1.36 (-4.51-1.78) |
| 3.10 - 5.40 | 1.97 (-1.53-5.47) | 0.30 (-2.90-3.51) |
| 5.50 - 108.00 | 0.30 (-3.58-4.17) | -0.05 (-3.64-3.54) |
| P trend | 0.56 | 0.82 |
| MEHP |  |  |
| 0.35 - 0.90 | ref | ref |
| 1.00 - 1.90 | -0.48 (-3.84-2.88) | -0.50 (-3.57-2.56) |
| 2.00 - 4.10 | 0.14 (-3.15-3.43) | -1.09 (-4.12-1.94) |
| 4.20 - 367.00 | -0.37 (-3.98-3.24) | -0.04 (-3.35-3.26) |
| P trend | 0.92 | 0.86 |
| MEHHP |  |  |
| 0.60 - 9.20 | ref | ref |
| 9.30 - 17.10 | 0.94 (-2.41-4.30) | 0.48 (-2.60-3.55) |
| 17.20 - 33.00 | 3.00 (-0.28-6.28) | 1.01 (-2.04-4.06) |
| 33.20 - 2830.00 | 4.06 (0.28-7.84) | 2.61 (-0.86-6.08) |
| P trend | 0.02 | 0.15 |
| MEOHP |  |  |
| 0.20 - 5.80 | ref | ref |
| 5.90 - 10.60 | -0.98 (-4.34-2.38) | 1.15 (-4.23-1.93) |
| 10.70 - 20.40 | 1.58 (-1.75-4.90) | 0.19 (-2.89-3.27) |
| 20.50 - 1610.00 | 2.77 (-0.99-6.53) | 1.61 (-1.85-5.07) |
| P trend | 0.10 | 0.33 |
| MECPP |  |  |
| 1.10 - 12.90 | ref | ref |
| 13.00 - 22.60 | 0.19 (-3.13-3.51) | -1.04 (-4.08-2.00) |
| 22.70 - 41.50 | 4.02 (0.72-7.33) | 1.94 (-1.15-5.03) |
| 41.60 - 2460.00 | 3.81 (-0.03-7.65) | 2.84 (-0.76-6.43) |
| P trend | 0.01 | 0.06 |
| ΣDEHP, μmol/L |  |  |
| 0.008 - 0.1007 | ref | ref |
| 0.1008 - 0.1827 | 0.18 (-3.11-3.47) | 0.08 (-2.95-3.12) |
| 0.1828 - 0.341 | 4.29 (0.95-7.63) | 1.93 (-1.19-5.06) |
| 0.343 - 24.419 | 3.21 (-0.65-7.08) | 2.83 (-0.75-6.41) |
| P trend | 0.03 | 0.08 |
| MCOP |  |  |
| 0.14 - 2.10 | ref | ref |
| 2.20 - 3.60 | -0.58 (-3.79-2.64) | -0.44 (-3.38-2.50) |
| 3.70 - 6.50 | 2.91 (-0.68-6.50) | 2.28 (-0.98-5.54) |
| 6.60 - 239.00 | 2.58 (-1.13-6.30) | 2.11 (-1.32-5.54) |
| P trend | 0.09 | 0.13 |
| MCNP |  |  |
| 0.14 - 1.50 | ref | ref |
| 1.60 - 2.60 | 1.51 (-1.80-4.82) | 1.21 (-1.81-4.24) |
| 2.70 - 4.70 | 1.52 (-1.95-4.99) | 1.28 (-1.91-4.47) |
| 4.80 - 91.60 | 2.24 (-1.38-5.86) | 2.50 (-0.81-5.82) |
| P trend | 0.25 | 0.15 |
| ^a^Adjusted for creatinine | | |
| ^b^Adjusted for creatinine, age, ethnicity, alcohol use, physical activity, smoking status, healthy eating index, dietary energy intake, hormone replacement therapy use, education, income, and history of diabetes, hypertension, dyslipidemia and cardiovascular diseases | | |
| Abbreviations used: MEP, monoethyl phthalate; MBP, monobutyl phthalate; MHBP, mono-hydroxybutyl phthalate; DBP, dibutyl phthalate; MiBP, mono-isobutyl phthalate; phthalate MHiBP, mono-hydroxyisobutyl phthalate; DiBP, di-isobutyl phthalate; MBzP, monobenzyl phthalate; MCPP, mono(3-carboxypropyl) phthalate; MEHP, mono(2-ethylhexyl) phthalate; MEHHP, mono(2-ethyl-5-hydroxyhexyl) phthalate; MEOHP, mono(2-ethyl-5-oxohexyl); DEHP, di(2-ethylhexyl)phthalate; MECPP, mono(2-ethyl-5-carboxypentyl) phthalate; MCOP, mono-carboxyoctyl phthalate; MCNP, mono-carboxynonyl phthalate | | |

**Table S4: Cross-sectional associations between phthalate biomarker concentrations and overweight and obesity compared to underweight/normal within the WHI, among participants with complete data on a reduced set of covariates (N=1,187).**

| **Phthalate Biomarkers, ng/mL** | **Overweight**  **OR (95% CI)^a^** | **Obese**  **OR (95% CI)^a^** |
| --- | --- | --- |
| MEP |  |  |
| 2.80 - 33.10 | ref | ref |
| 33.20 - 67.90 | 0.84 (0.56 - 1.27) | 0.69 (0.44 - 1.10) |
| 68.10 - 159.00 | 0.85 (0.55 - 1.29) | 0.68 (0.43 - 1.10) |
| 161.00 - 26000.00 | 0.78 (0.51 - 1.21) | 0.62 (0.38 - 1.01) |
|  |  |  |
| ΣDBP, μmol/L |  |  |
| 0.002 - 0.065 | ref | ref |
| 0.0652 - 0.132 | 0.99 (0.67 - 1.45) | 1.09 (0.70 - 1.71) |
| 0.133 - 0.264 | 0.98 (0.64 - 1.49) | 1.44 (0.90 - 2.30) |
| 0.265 - 18.255 | 1.08 (0.67 - 1.72) | 1.19 (0.71 - 2.01) |
|  |  |  |
| ΣDiBP, μmol/L |  |  |
| 0.002 - 0.0057 | ref | ref |
| 0.006 - 0.0123 | 0.89 (0.60 - 1.32) | 0.96 (0.61 - 1.50) |
| 0.0124 - 0.0247 | 1.34 (0.88 - 2.05) | 1.7 (1.06 - 2.72) |
| 0.0248 - 1.339 | 1.95* (1.21 - 3.14) | 1.73 (1.01 - 2.96) |
|  |  |  |
| MBzP |  |  |
| 0.40 - 5.90 | ref | ref |
| 6.00 - 12.00 | 1.14 (0.76 - 1.70) | 2.44* (1.52 - 3.93) |
| 12.10 - 22.20 | 0.99 (0.65 - 1.49) | 1.61 (0.98 - 2.65) |
| 22.30 - 3590.00 | 1.41 (0.88 - 2.25) | 2.31* (1.34 - 3.99) |
|  |  |  |
| MCPP | |  |
| 0.14 - 1.70 | ref | ref |
| 1.80 - 3.00 | 1.14 (0.76 - 1.72) | 1.09 (0.67 - 1.75) |
| 3.10 - 5.40 | 1.39 (0.91 - 2.14) | 1.91* (1.19 - 3.08) |
| 5.50 - 108.00 | 1.43 (0.88 - 2.32) | 1.49 (0.87 - 2.54) |
|  |  |  |
| ΣDEHP, μmol/L |  |  |
| 0.008 - 0.1007 | ref | ref |
| 0.1008 - 0.1827 | 1.57 (1.07 - 2.32) | 1.51 (0.95 - 2.40) |
| 0.1828 - 0.341 | 2.00* (1.31 - 3.07) | 2.65* (1.65 - 4.27) |
| 0.343 - 24.419 | 2.36* (1.44 - 3.86) | 3.29* (1.92 - 5.62) |
|  |  |  |
| MCOP |  |  |
| 0.14 - 2.10 | ref | ref |
| 2.20 - 3.60 | 1.71* (1.16 - 2.52) | 1.62 (1.03 - 2.54) |
| 3.70 - 6.50 | 1.93* (1.26 - 2.98) | 2.61* (1.62 - 4.20) |
| 6.60 - 239.00 | 2.40* (1.51 - 3.82) | 2.54* (1.51 - 4.27) |
| MCNP |  |  |
| 0.14 - 1.50 | ref | ref |
| 1.60 - 2.60 | 1.35 (0.92 - 2.00) | 1.75 (1.11 - 2.78) |
| 2.70 - 4.70 | 1.17 (0.77 - 1.80) | 2.14* (1.33 - 3.45) |
| 4.80 - 91.60 | 1.77 (1.13 - 2.77) | 2.25* (1.35 - 3.76) |
